# An in vitro study of the fixed edentulous implant guide using a novel approach to edentulous guided surgery using a developed two part screw

**DOI:** 10.1038/s41405-025-00361-5

**Published:** 2026-01-09

**Authors:** Adam B. Nulty

**Affiliations:** University of Leeds, Leeds, UK

**Keywords:** Dental implants, Fixed prosthodontics

## Abstract

**Introduction:**

Accurate implant placement in edentulous patients remains a clinical challenge, particularly when using mucosa-supported guides, which are prone to positional errors.

**Aims:**

To evaluate the accuracy of implant placement using the Fixed Edentulous Implant Guide (FEIG), a screw-retained guide system, compared with a conventional mucosa-supported guide in an edentulous mandible model.

**Materials and methods:**

Ten anatomical mandibular models with simulated mucosa and bone were used. Forty dummy implants were placed using either the FEIG system or a conventional guide. Implant positions were virtually planned, and deviations were assessed by comparing the planned and actual positions using STL-based alignment and a custom measurement algorithm.

**Results:**

The FEIG system demonstrated significantly lower mean deviations at the coronal (0.45 ± 0.15 mm) and apical (0.28 ± 0.13 mm) levels compared with the conventional guide (1.22 ± 0.61 mm and 0.89 ± 0.39 mm, respectively; *p* < 0.001). Angular deviation was lower for the FEIG (1.99° ± 0.98) but not statistically significant (*p* = 0.081).

**Conclusions:**

The FEIG method significantly improved the accuracy of implant placement in vitro compared to a conventional edentulous guide. Further in vivo studies are warranted to confirm its clinical applicability.

## Introduction

Accurate implant placement in fully edentulous patients—those with complete loss of all natural teeth—remains a clinical challenge [1]. Mucosa-supported surgical guides are widely used in guided surgery for these cases but are prone to displacement and distortion, leading to significant deviations in implant position [2]. This is especially problematic in flapless protocols where visibility and tactile feedback are limited [3]. The Fixed Edentulous Implant Guide (FEIG) system investigated in this study introduces a screw-retained method of stabilising the surgical guide via a two-part orthodontic fixation screw, intended to improve guide stability and implant placement accuracy. This innovative approach aims to enhance the precision of implant placement by addressing the common issues associated with mucosa-supported guides, such as displacement and distortion [4].

Implant surgery in edentulous patients must often balance the desire for minimal invasiveness with the need for precision [5]. Traditional approaches such as flap raising increase morbidity and may delay healing [6]. Conversely, mucosa-supported guides used in flapless surgery can result in angular and linear deviations, particularly in the absence of hard-tissue reference points [2]. The FEIG approach aims to bridge this gap by rigidly referencing both hard and soft tissues during guide placement. In addition to enhancing stability, the FEIG system’s reliance on a screw-retained mechanism may also facilitate more accurate intra-operative adjustments, allowing for real-time corrections during the surgical procedure. This adaptability is particularly crucial in edentulous cases where anatomical variations can lead to unexpected challenges. Furthermore, emerging technologies such as real-time imaging and soft tissue navigation systems can complement the FEIG approach, providing surgeons with more comprehensive feedback on implant positioning and soft tissue interactions [7]. By integrating these advanced imaging techniques, the potential for achieving optimal implant placement accuracy may be significantly improved, thereby reducing the risk of postoperative complications and enhancing patient outcomes in guided surgeries.This study highlights the importance of utilising innovative techniques, such as the FEIG system, to improve the accuracy and outcomes of flapless implant surgeries in edentulous patients.

### Background

Approximately 6% of the population in England, Wales and Northern Ireland is edentulous [8, 9]. Globally, complete edentulism affects over 150 million people, representing a significant functional and psychosocial burden [10–13]. Edentulism leads to reduced mastication, altered speech and changes in facial appearance and support [14]. Conventional treatment options have historically included removable complete dentures, but fixed implant-supported prostheses offer superior function and patient satisfaction [15]. The McGill Consensus (2002) recommends mandibular two-implant overdentures as the minimum standard of care [16, 17].

Despite the functional benefits of implants, complications may arise from misplacement due to inadequate guide stability or surgical visibility [18, 19]. Angular deviations, coronal displacement and apical divergence can all compromise outcomes [20]. These errors are influenced by the guide design, fixation method and whether the surgery is performed with or without a flap [21]. The use of large flaps, while improving visibility, increases the risk of soft tissue trauma, delayed healing, infection and morbidity [22, 23].

Alternative solutions are necessary for elderly or medically compromised patients who may not tolerate invasive surgery. Studies have shown that implant-retained over-dentures improve oral health-related quality of life [19]. At the same time, there is recognition that many patients - especially the elderly - may be reluctant to undergo extensive procedures [24]. In such cases, minimally invasive, flapless guided surgery using stable reference guides could improve both clinical outcomes and patient acceptance.

As the demand for less invasive options grows, the integration of digital technologies in implant surgery continues to gain traction, offering a promising avenue for enhancing surgical precision [25]. Computer-guided surgery, utilising advanced imaging techniques such as cone-beam computed tomography (CBCT), allows for meticulous preoperative planning and real-time feedback during procedures [26]. This approach not only minimises the risk of complications associated with traditional methods but also aligns with the increasing preference among patients for procedures that prioritise comfort and reduced recovery times [27]. Furthermore, studies have reported that flapless computer-guided techniques can achieve high implant survival rates while maintaining minimal soft tissue trauma [28], thus supporting the notion that innovative methodologies like the FEIG system could further optimise patient outcomes in this evolving field of dental surgery [29]. By fostering a collaborative environment that combines technological advancements with novel surgical techniques, practitioners can better address the complexities of edentulous cases and improve overall patient satisfaction [30, 31].

### Aims

The aim of this study was to measure the accuracy of implant placement using the FEIG method in comparison to a conventional mucosa-supported surgical guide in an in vitro edentulous mandible model. The FEIG system uses two-part orthodontic fixation screws to create a stable reference between CBCT imaging and intraoral scanning.

Implant planning was carried out using SMOP guided surgery software. Rather than comparing pre- and post-operative CBCTs, implant positions were exported as 3D STL models and the final implant positions were recorded using scan bodies. The planned and actual implant STL positions were aligned and compared using a custom algorithm developed by the Leeds School of Dentistry to calculate trueness and precision [32, 33].

Null hypothesis (H_o_): There is no difference in the implant placement error between the FEIG system and a conventional tissue-borne surgical guide in an in vitro edentulous mandible simulation.

## Materials and methods

### Sample size

A power calculation based on preliminary in vitro data was performed using the BioMATH sample size calculator, with 80% power and a significance level of *p* < 0.05. This determined that a sample size of five models per group would be sufficient. All equipment was calibrated prior to the start of the study. To assess placement accuracy and isolate the sources of error, each step of the digital workflow was standardised and documented.

### Anatomical model fabrication

**Fig. 1:**
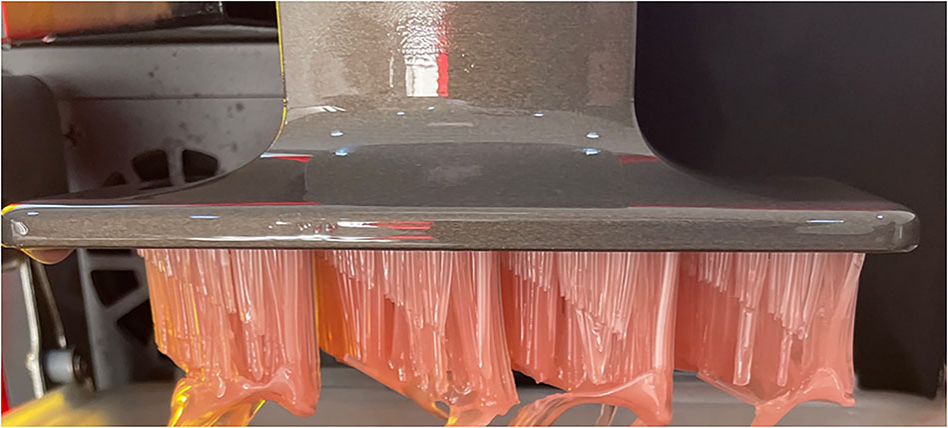
3D Printed Gingiva to attach to the Artificial Mandibles.

**Fig. 2:**
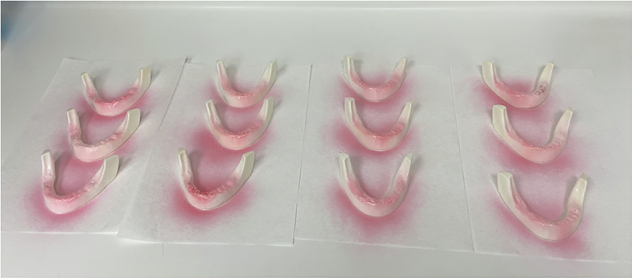
Artificial Mandibles are sprayed with adhesive to bond the artificial gum layer.

To simulate clinical conditions, a more biologically representative lower jaw model was created. Artificial D2-density mandibles (Model #1522-42, Sawbones, Vashon Island, WA, USA) were used as the bone base. The mandibular model surface was optically scanned (Up3D 300e, Up3D Dental, Seoul, Korea) and a soft tissue layer was designed by extruding the surface geometry by 1.5 mm—the average mucosal thickness in edentulous mandibles [9]. This gingival layer was 3D printed using Asiga Dental Gum resin (Asiga, Australia) and bonded to the mandible using adhesive spray (Figs. 1 and 2) [34].

The model design simulated an extensively resorbed edentulous ridge, classified as Atwood Class V, characterised by a smooth, flattened residual ridge.35 While the model does not fully replicate the clinical variability of patient-specific bone and mucosa, it provides a consistent standard for comparative accuracy measurements.

**Fig. 3:**
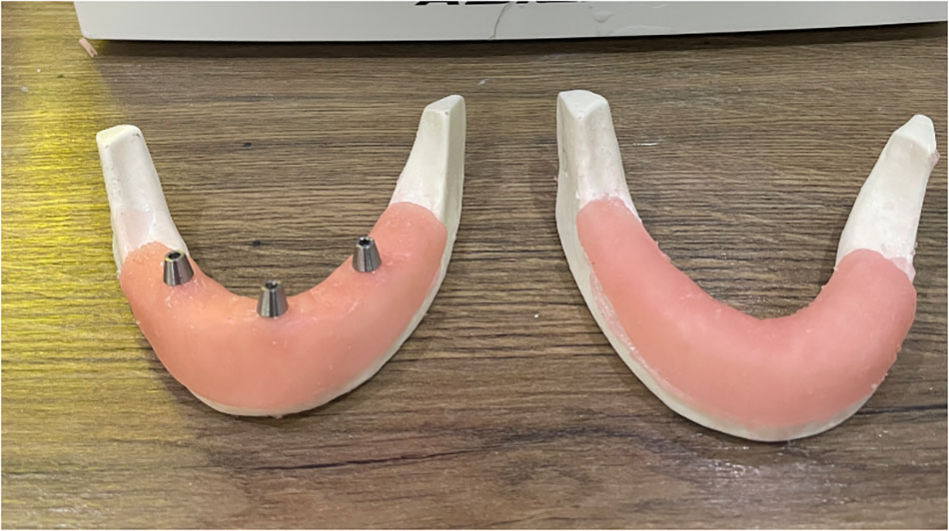
Artificial Bone Model Type A - With Novel Two Part Screws and Type B - Without screws.

Five models were prepared with the FEIG system, and five with a conventional mucosa-supported guide. The FEIG models incorporated a set of three stainless steel two-part fixation screws (length: 10 mm, diameter: 1.6 mm; custom-manufactured) arranged in a triangular configuration. These screws anchored into the bone and acted as fixed reference markers in both CBCT imaging and intraoral scanning (Fig. 3). The two-part screw featured a coronal component that interfaced with the guide during seating, ensuring repeatable positioning.

**Fig. 4:**
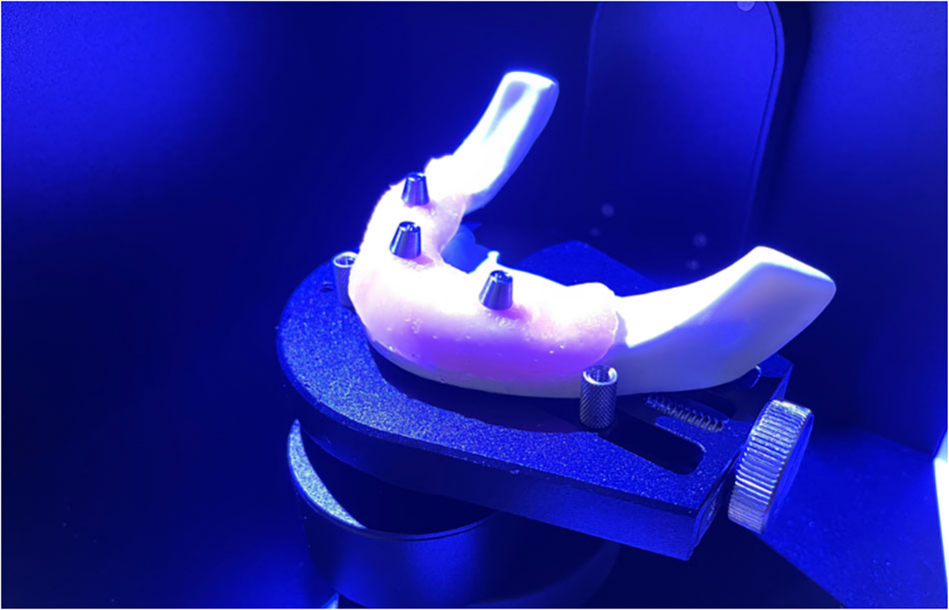
Each Model was scanned with an Up3D 300e lab light scanner.

**Fig. 5:**
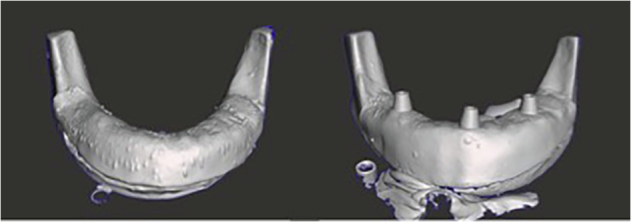
The 3D STL scan of each model type.

Each model was labelled and scanned with a laboratory scanner (Up3D 300e) to generate a high-resolution STL file for planning and analysis (Figs. 4 and 5) [14].

### Virtual planning and CBCT acquisition

**Fig. 6:**
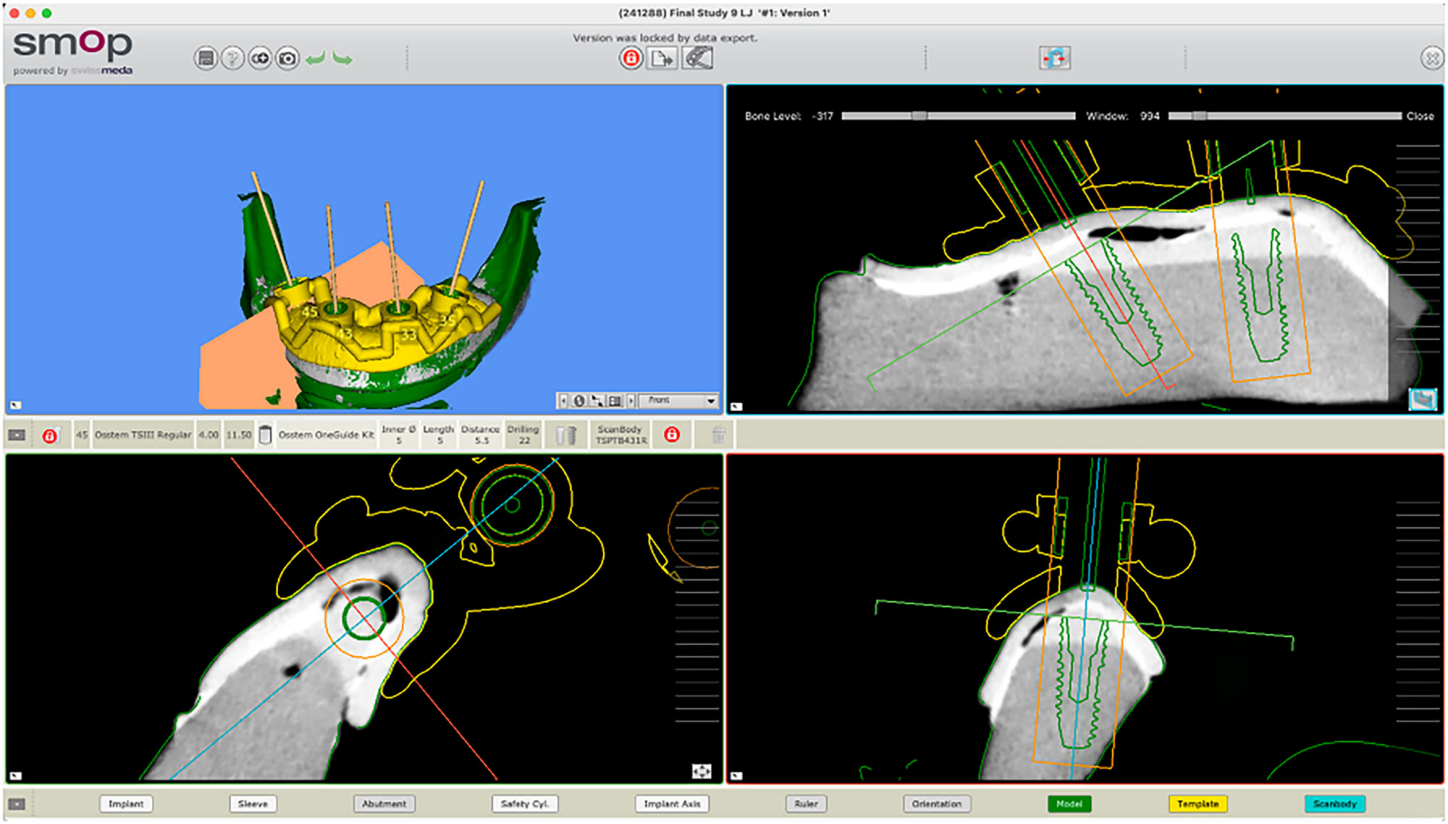
The Swissmeda SMOP Implant Planning.

CBCT scans were acquired using a Carestream CS 8100 3D system (Carestream Dental, Atlanta, USA) with a voxel size of 0.2 mm, standard protocol for edentulous lower arch imaging. The STL file from the lab scan and the DICOM data were imported into SMOP guided surgery software (Swissmeda, Zurich, Switzerland; version 2.11), where four dummy implants (4.0 mm diameter × 10.0 mm length) were virtually planned for each model (Fig. 6).

### Guide fabrication

Surgical guides were designed using SMOP and exported as STL files. Guides were printed using an Asiga Max UV 3D printer (Asiga, Australia) with a 25 µm layer thickness using NextDent SG Guide resin (NextDent, The Netherlands). Each guide was post-processed according to manufacturer instructions: ultrasonic washing in isopropyl alcohol, followed by curing under UV light. The FEIG guides included fixation ports to lock onto the pre-placed screw heads, while the conventional guides were designed as soft-tissue-supported templates with no additional retention features.

### Implant placement protocol

Forty dummy implants were placed in ten mandible models (four per model), using the Osstem OneGuide Sleeveless Drill Kit (Osstem, Seoul, South Korea). Gingival tissue was removed at the osteotomy sites using a standard tissue punch.

**Fig. 7:**
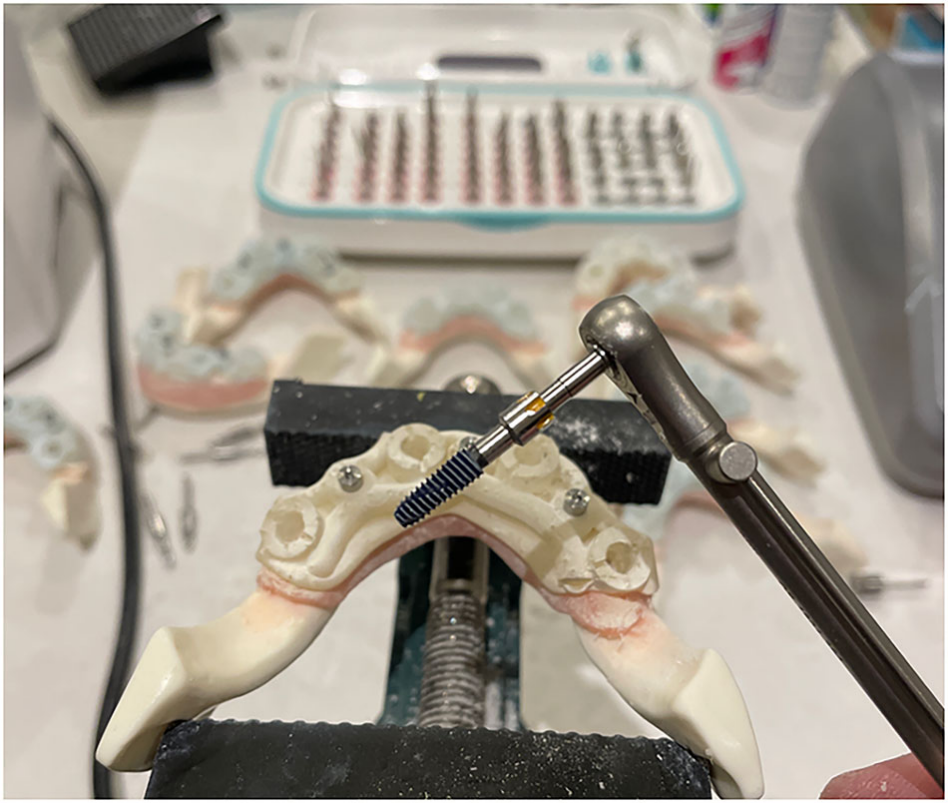
The test block osteotomies and dummy implant placement.

**Fig. 8:**
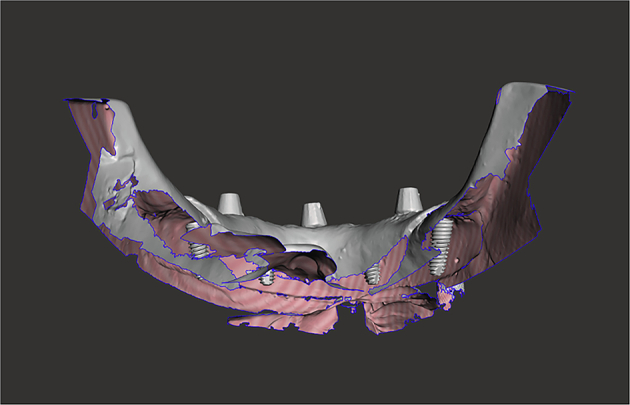
An STL Export of the Original Impression Data and the Export of the Planned Virtual Implant STLs.

**Fig. 9:**
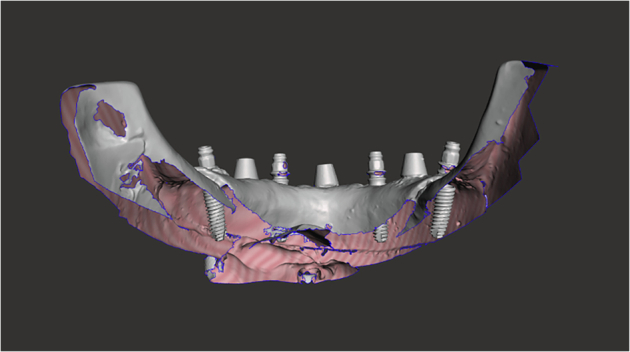
An STL export of the post-placement impression data. The image shows the calculated implant position below the gingiva calculated from the Scan Bodies shown above the gingiva.

A fully guided, sequential drilling protocol was followed: pilot drills were used first, followed by successive diameter-specific twist drills as per the Osstem OneGuide protocol (e.g. 2.0 mm, 2.8 mm, 3.5 mm, final 4.0 mm), all through the guide sleeves. Drills were operated at 1500 rpm with saline irrigation (insert actual value if different). Dummy implants were seated using guide mounts to ensure coaxial insertion (Figs. 7, 8, 9).

Conventional guides (non-FEIG) were fabricated using the same protocol but seated directly on the gingival surface without bone-level fixation.

### Accuracy analysis and implant position calculation

After implant placement, the models were scanned using the same Up3D 300e lab scanner. Scan bodies were attached to each implant to facilitate alignment and position tracking. Planned implant STL files and post-placement STL files were aligned using Exocad DentalCAD software (Exocad GmbH, Darmstadt, Germany) and exported for analysis.

Each implant was labelled by model number and position (A–D) and categorised as pre-planned or post-placement. A custom in-house C++ programme was used to compute positional changes between corresponding STL meshes.

Each specific implant STL was then exported with a nomenclature based on the model number and position from A to D along with whether it was a pre-planned position or a post-placed position Table 1. This process resulted in a folder with identical implant STLs apart from the 3D XYZ coordinates of each STL. Through this method the following changes in 3D position were calculated:

- Deviation of the centre of the coronal aspect of the implant in terms of X, Y and Z
- Deviation of the centre of the apical aspect of the implant in terms of X, Y and Z
- Vertical angulation change.

**Table 1:** Test data recorded from comparison of implant positions.

BLOCK REPEAT	Position	Coronal Deviation A (mm)	Apical Deviation B (mm)	Angle Error (Degrees)
1	a	0.76	0.50	2.22
1	b	0.43	0.41	1.15
1	c	0.53	0.43	1.00
1	d	0.55	0.41	3.06
2	a	0.55	0.25	2.19
2	b	0.47	0.19	2.32
2	c	0.54	0.38	2.11
2	d	0.62	0.50	2.58
3	a	0.34	0.23	2.15
3	b	0.36	0.33	0.69
3	c	0.32	0.16	1.38
3	d	0.11	0.16	0.21
4	a	0.23	0.31	2.26
4	b	0.33	0.14	1.40
4	c	0.28	0.09	1.59
4	d	0.60	0.36	4.62
5	a	0.36	0.09	2.27
5	b	0.64	0.17	3.35
5	c	0.44	0.18	1.44
5	d	0.57	0.31	1.83
6	a	1.03	1.13	1.97
6	b	2.59	1.75	6.96
6	c	1.21	1.45	2.25
6	d	2.84	1.56	7.25
7	a	1.89	0.70	6.40
7	b	1.38	1.10	2.40
7	c	0.92	0.92	0.31
7	d	1.29	0.67	3.24
8	a	0.72	0.54	1.23
8	b	1.01	0.68	2.24
8	c	0.73	0.54	1.13
8	d	0.73	0.69	1.61
9	a	0.98	0.61	3.81
9	b	1.07	0.93	2.58
9	c	1.45	1.53	1.37
9	d	1.08	0.67	3.53
10	a	0.28	0.31	1.41
10	b	0.84	0.64	1.34
10	c	1.28	0.81	3.34
10	d	1.14	0.65	3.00

A custom made C++ programme was then used to calculate the direct XYZ positional changes of the STLs as described by the author in a previous publication [8].

The *X*, *Y*, *Z* values are the absolute positions recorded. Deviation A and Deviation B are calculated as sqrt(X*X + Y*Y + Z*Z) and are the key linear deviation magnitudes used for statistical analysis of the results.

Once the comparison was completed the numerical analysis was added to the clipboard and recorded into a table.

### Ethics declaration

As this was an in vitro study conducted on a 3D-printed edentulous model rather than human or animal subjects, ethical approval and informed consent were not required. No patient-identifiable data or clinical interventions were involved.

## Results

### Statistical analysis

Statistical analysis was performed using SPSS 27 statistical analysis software [36]. The statistics were performed in four separate groups as each model has fours implants. The data from these four positions were not independent as they had the same guide sitting in the same position. The results were therefore distinguished into four data sets in positions A, B, C and D for the comparison to the repeats of the first model data with the intention to analyse with a *t*-test.

### Data obtained

**Fig. 10:**
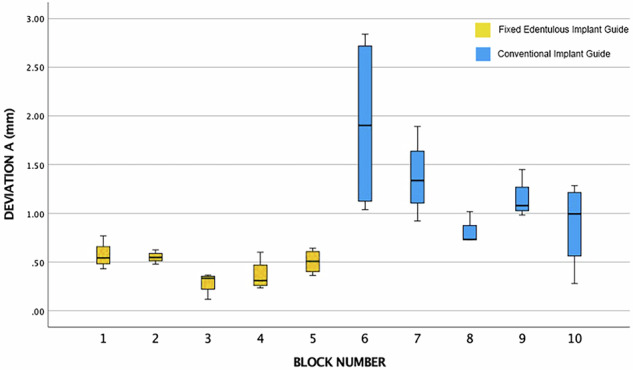
Box Plot of the Data Output Categories for the Overall Coronal Deviation **A**. Blocks 1–5 are with the Fixed Edentulous Implant Guide. Blocks 6–10 are with a conventional edentulous implant guide.

**Fig. 11:**
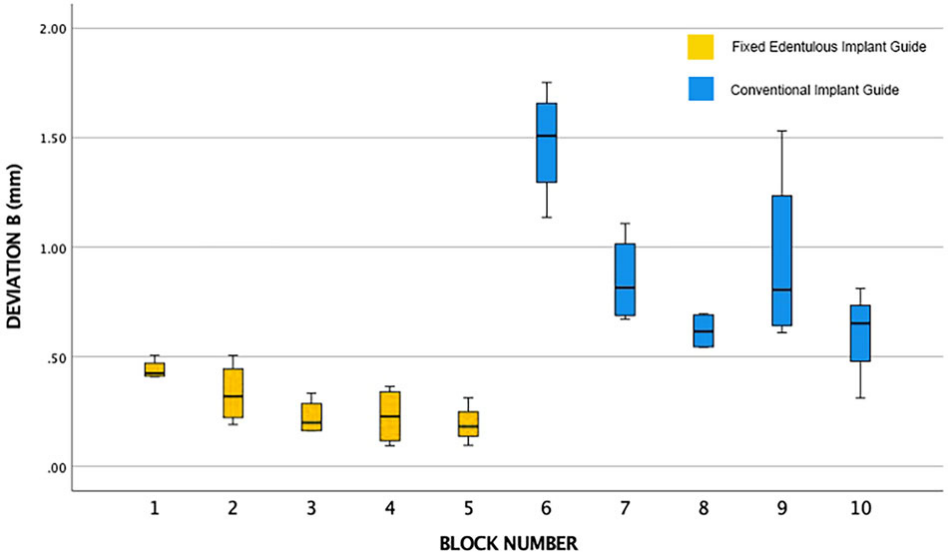
Box Plot of the Data Output Categories for the Overall Apical Deviation **B**. Blocks 1–5 are with the Fixed Edentulous Implant Guide. Blocks 6–10 are with a conventional edentulous implant guide.

**Fig. 12:**
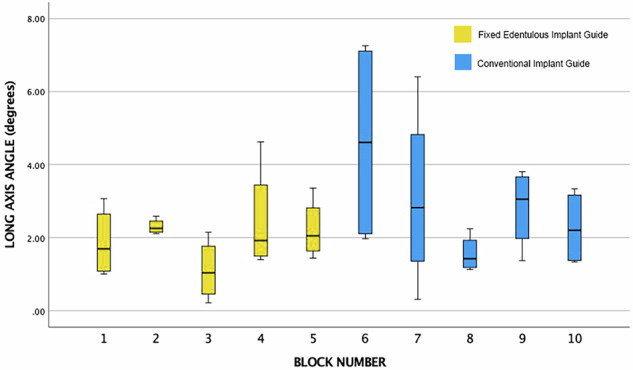
Box plot of the data output categories for the overall vertical angular deviation. Blocks 1–5 are with the fixed edentulous implant guide. Blocks 6–10 are with a conventional edentulous implant guide.

A box plot of the data output categories as given by the batch calculator tool is shown in Figs. 10, 11 and 12.

The box plots are not presented as two groups due to the inherent structure and dependencies within the data. Given that the study involved four separate groups, each with four implants, the data from these four positions are not independent, as they share the same guide sitting in the same position. This interdependence necessitates the segregation of results into distinct datasets corresponding to each block, rather than amalgamating them into two broader groups. This categorisation enables a more precise comparison to the repeats of the block data. The intention to analyze with a *t*-test further underscores the need for maintaining the integrity of these individual datasets, as pooling them into two groups could obscure the subtleties and variances inherent to each position, potentially leading to inaccurate or misleading statistical inferences.

### Statistical analysis of data sets

The group statistics reveals that the Novel Method 1 has a lower mean for each type of deviation, be it overall deviation coronally, apically, or the angulation change, as shown in Table 2.

**Table 2:** Independent Samples *T*-Test: Novel Method 1 versus Regular Method 2.

Method 1 (Screw) and Method 2 (No Screw)				
	Method	Mean	Std. Deviation	Std. Error Mean
Deviation A (mm)	1	0.45	0.15	0.03
	2	1.22	0.61	0.13
Deviation B (mm)	1	0.28	0.13	0.02
	2	0.89	0.39	0.08
Long Axis Angulation Deviation (Degrees)	1	1.99	0.98	0.22
	2	2.87	1.95	0.43

Furthermore, the mean for the overall deviation A and B are both over half for the novel method.

Regarding the independent samples *t*-test, Levene’s test for equality of variances is less than 0.05 for each of the deviation types, which means that the variability in each of the types of deviation between each method is not the same and is significantly different.

The *t*-test results as shown in Table 3, reveal that the significance is less than than 0.05 for each comparison (*p* < 0.001), and a statistically significant difference between the two methods is concluded. Furthermore, the differences between condition means are not likely due to chance, and the novel method is significantly more accurate in terms of coronal and apical deviation. However the same is not true for the overall angulation error (*p* = 0.081). Here the *t*-test shows that there is no statistically significant difference between the two methods. The differences between condition means are likely due to chance.

**Table 3:** Main data descriptive statistics.

Independent Samples Test										
		Levene’s Test for Equality of Variances				*t*-Test for Equality of Means			95% Confidence Interval for the Difference	
		*F*	Sig.	*t*	df	Sig. (2-tailed)	Mean Difference	Std. Error Difference	Lower	Upper
Deviation A	Equal Variances assumed	8.15	0.00	−5.46	38	0.00	−0.77	0.14	−1.05	−0.48
	Equal Variances not assumed			−5.46	21.58	0.00	−0.77	0.14	−1.06	−0.47
Deviation B	Equal Variances assumed	16.22	0.00	−6.54	38	0.00	−0.61	0.09	−0.80	0.42
	Equal Variances not assumed			−6.54	23.10	0.00	−0.61	0.09	−0.80	−0.41
Long Axis Angulation Deviation	Equal Variances assumed	5.38	0.02	−1.79	38	0.08	−0.87	0.48	−1.86	0.11
	Equal Variances not assumed			−1.79	28.05	0.08	−0.87	0.48	−1.87	0.12

## Discussion

The FEIG guide yielded significantly lower linear deviations at both the coronal and apical aspects of implant placement compared to the conventional mucosa-supported guide, with mean deviations of 0.45 ± 0.15 mm and 0.28 ± 0.13 mm, respectively. In contrast, the conventional guide produced mean deviations of 1.23 ± 0.60 mm (coronal) and 0.90 ± 0.40 mm (apical). Angular deviation was also lower with the FEIG method (1.99° ± 0.98) compared to the control (2.87° ± 1.95), though this difference was not statistically significant (*p* = 0.081).

These findings are consistent with the values reported by Behneke et al., who assessed guided surgery in similar in vitro settings [37]. More recent reviews also highlight the reduced accuracy of mucosa-borne guides due to soft tissue compression and movement during scanning and surgery [2]. The use of bone-level screw fixation in the FEIG system appears to mitigate these factors and may account for the improved trueness observed in this study.

The implications of adopting the FEIG system extend beyond mere accuracy in implant placement; they also underscore a potential shift in clinical practice towards more patient-centred approaches in edentulous surgeries. By minimising the need for flap procedures and leveraging screw-retained stability, practitioners may not only enhance surgical outcomes but also improve patient comfort and satisfaction, which is paramount in the context of increasing patient reluctance toward invasive treatments. Furthermore, as the field advances, the integration of artificial intelligence and machine learning into the planning and execution phases of guided surgeries could further refine the accuracy and predictability of outcomes, as highlighted by recent studies advocating for the use of advanced imaging and digital workflows in surgical settings [38, 39]. This evolution in technology, coupled with the FEIG’s innovative design, could pave the way for a new standard of care that prioritises both clinical efficacy and the overall patient experience, ultimately leading to enhanced quality of life for those affected by edentulism. The findings suggest that the FEIG system not only improves implant placement accuracy but may also enhance overall patient satisfaction and quality of life in edentulous individuals, aligning with recent evidence on the benefits of implant-supported prostheses [40].

The measurement approach using STL file comparison and a custom alignment algorithm was validated internally and has been used in previous research. It allowed for direct quantification of 3D positional changes in XYZ space between planned and actual implant locations. Precision was achieved by maintaining consistent scan conditions and using labelled, high-resolution STL exports.

The novelty of the FEIG system lies in its use of a two-part bone-anchored fixation screw design. This allows the surgical guide to be referenced to both hard and soft tissues during digital planning and clinical placement. To our knowledge, no previous guide design has employed this specific mechanism for fully edentulous patients, which positions FEIG as a potentially significant advancement in edentulous guided surgery. Moreover, the potential for integrating digital workflows with augmented reality (AR) technologies presents an exciting frontier for enhancing the FEIG system’s capabilities. By overlaying real-time imaging data onto the surgical field, AR could provide surgeons with immediate visual guidance, ensuring that implant placement adheres to the planned trajectory even in complex anatomical scenarios [41]. This could further mitigate the challenges posed by soft tissue variations and enhance the precision of flapless procedures, aligning with findings that emphasise the importance of advanced imaging in improving surgical outcomes [42]. Additionally, the incorporation of machine learning algorithms into the planning phase could refine the predictive accuracy of implant outcomes, allowing for personalised treatment strategies that accommodate individual anatomical differences. As these technologies evolve [43], they not only promise to elevate the standards of care in edentulous surgeries but also enrich the patient experience by minimising invasiveness and promoting faster recovery times [44].

### Limitations

This study was conducted in vitro using standardised anatomical mandible models with printed gingiva. While this model provided a reproducible platform for comparison, it cannot fully replicate the viscoelastic behaviour of living soft tissues. Prior studies have shown that mucosal displacement and hydration can affect guide seating in vivo, potentially influencing clinical accuracy [45, 46].

The study focused on immediate implant placement accuracy and did not evaluate clinical outcomes such as osseointegration, implant survival, or prosthetic complications. These aspects would need to be addressed in a long-term in vivo study.

Another limitation concerns guide design. The guides used in this study incorporated a polymer-based drill sleeve without a metal insert. While this design simplified fabrication, it introduces the potential for thermal or material contamination, particularly in surgical environments. Additionally, the guide tube length and fit were limited by the dummy implant system used, and these factors were not controlled in the study. Both issues may influence accuracy and should be explored further in future research.

The sample size, while sufficient for detecting significant differences in linear deviation, may have limited the ability to detect differences in angular deviation. Increasing the number of test models could improve statistical power in future trials.

### Future directions

The findings of this study support further investigation of the FEIG method in vivo. A clinical trial involving flapless full-arch rehabilitation would provide a more definitive assessment of safety, morbidity and long-term outcomes. Additionally, future work should compare FEIG to other emerging guide designs—such as pin-retained or bone-supported templates—and explore performance across varying edentulous ridge anatomies, particularly different Atwood classifications [35].

Addressing these factors will help validate the FEIG system’s potential to improve accuracy in guided surgery while enabling minimally invasive treatment options for edentulous patients.

### Clinical implications and novel contribution

The results of this study suggest that the FEIG may enable more accurate placement of implants in edentulous patients compared to conventional mucosa-supported guides. This level of accuracy supports the potential for wider adoption of flapless implant protocols, especially in patients with medical contraindications to conventional surgery. Because FEIG improves placement precision without the need for soft tissue reflection, it may help reduce surgical morbidity when sufficient bone volume is present.

This aligns with current consensus recommendations, such as those of the British Society of Prosthodontics [47], which advocate a minimum of two implants to support mandibular overdentures as standard care in edentulous patients. Improved placement accuracy through FEIG could facilitate this approach for a broader patient population by making flapless implant delivery safer and more predictable [48].

The key novel feature of the FEIG system is its screw-retained fixation, which contrasts with the soft-tissue support used in standard guides. The design uses a unique two-part fixation screw, allowing reproducible referencing between CBCT and surface scans. No previous guide system to our knowledge has incorporated this configuration in fully edentulous cases, marking a distinct contribution to the field of digital implant surgery. Emerging literature highlights the importance of fixation in guided accuracy, further validating the focus on rigid stabilisation in FEIG [2]. As the landscape of dental implantology evolves, the integration of patient-specific factors into the planning and execution of surgeries becomes increasingly critical. Tailoring the FEIG system to accommodate individual anatomical variations could enhance its efficacy, particularly for patients with unique ridge morphologies or those exhibiting significant resorption patterns. Furthermore, the incorporation of advanced imaging modalities, such as artificial intelligence-driven analysis of CBCT scans, may allow for more precise predictions of implant placement outcomes, thereby addressing the inherent variability in soft tissue response and bone quality observed in edentulous patients [49]. This approach not only aligns with the growing emphasis on personalised medicine but also underscores the necessity of developing adaptable surgical protocols that can respond dynamically to the complexities of each case. Ultimately, such innovations could pave the way for a more holistic understanding of implant success, extending beyond mere placement accuracy to encompass broader patient health and satisfaction outcomes.

Future in vivo research is required to evaluate clinical outcomes, particularly in cases with varying ridge anatomies or Atwood classifications, and to confirm whether FEIG’s advantages persist in dynamic soft-tissue environments.

## Conclusion

The FEIG guide demonstrated significantly improved implant placement accuracy in vitro compared to a conventional mucosa-supported guide, with reduced coronal and apical deviations. These results support the potential clinical benefit of a screw-retained reference system for guided implant placement in fully edentulous cases. Further in vivo studies are warranted to confirm its performance in clinical settings.

## Data Availability

All data generated or analysed during this study are included in the published article and its supplementary materials. Additional datasets may be made available from the corresponding author upon reasonable request.
